# Supplementary figures and images for: Leptin-a mediates transcription of genes that participate in central endocrine and phosphatidylinositol signaling pathways in 72-hour embryonic zebrafish (Danio rerio)
Source: PeerJ. 2019 May 3;7:e6848. doi: 10.7717/peerj.6848 (PMC6501765; doi:10.7717/peerj.6848)

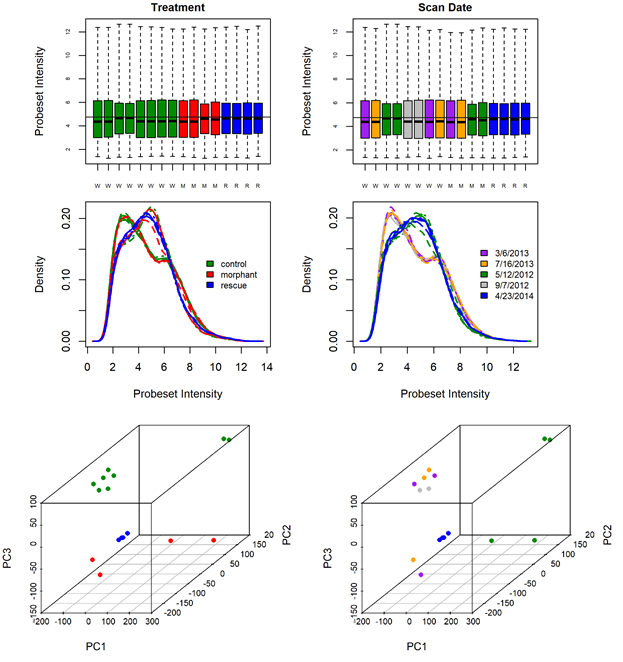

Supplement: Figure S1 — ([Top Panel]) Box-and-whisker plots illustrate the distribution of all 75,212 log2 probeset intensities derived from each microarray; samples are colored by treatment (top) and scan date (bottom). The horizontal black line represents the consensus mean intensity. Shaded areas span the distance between upper and lower quartiles. Sample medians are represented by horizontal black notches within each box on the plot. Outliers are indicated by dashed lines. “W” = uninjected control, “M” = lepa morphant, “R” = lepa rescue. [Middle Panel] Probeset signal density histograms represent the signal density versus log2 intensity relationship for 75,212 probesets on separate microarrays (n = 16); plots are colored by treatment (left) and scan date (right). [Bottom Panel] Principle component analyses generated from 75,212 probeset expression estimates are colored by treatment (left) and scan date (right). Each point represents one microarray sample where points closer in spatial orientation have comparable variable features. [file peerj-07-6848-s001.png]

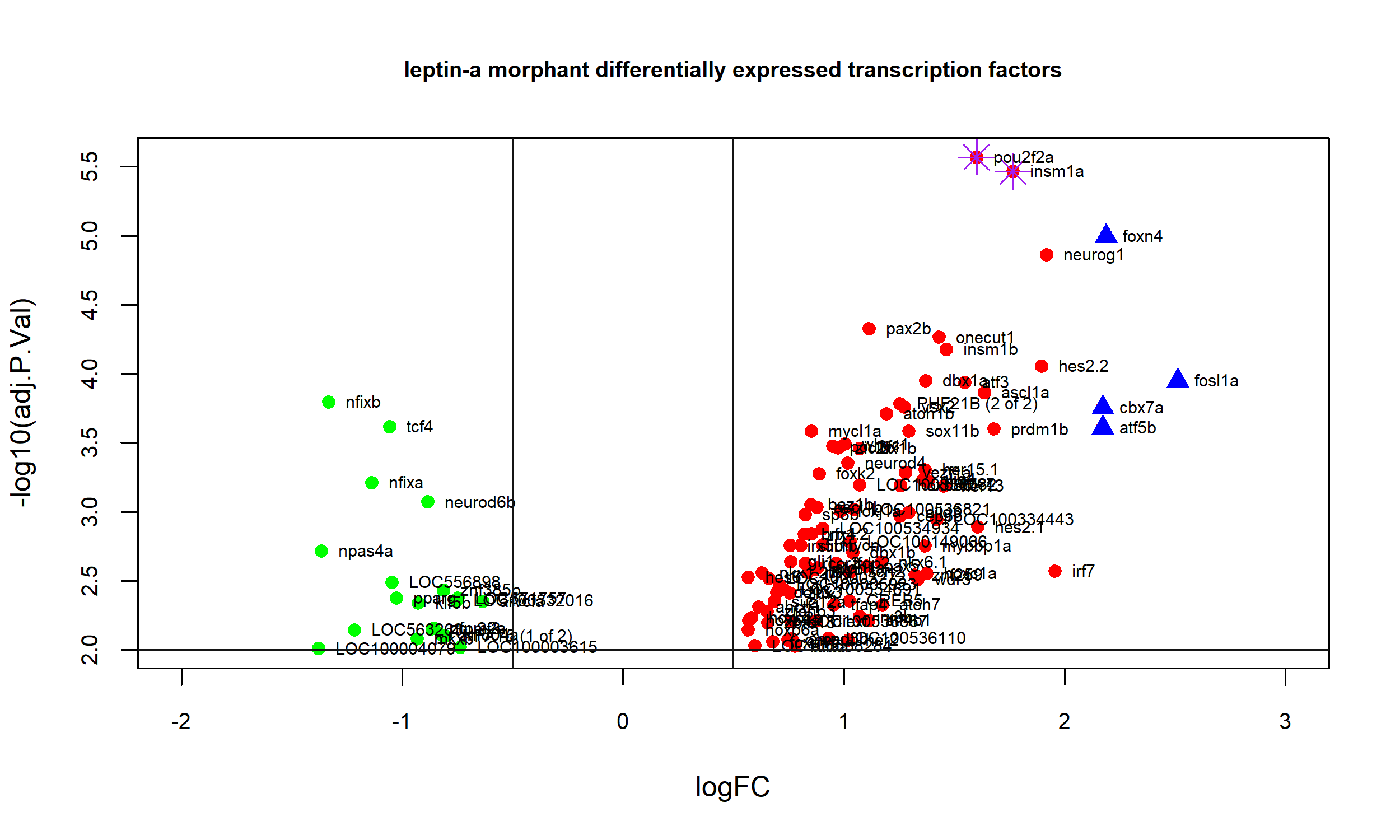

Supplement: Figure S2 — Volcano plot representing 112 differentially expressed transcription factors from the lepa knockdown compared to control zebrafish embryos. Green and red points denote down- and upregulated genes, respectively. Blue triangles represent differentially expressed transcription factors with log2 fold changes >2. Purple stars represent transcription factors that rank among the top-10 differentially expressed genes in lepa knockdown compared to control treatments. Horizontal and vertical axes represent the fold change and p.value selection criterions for differentially expressed genes. [file peerj-07-6848-s002.png]

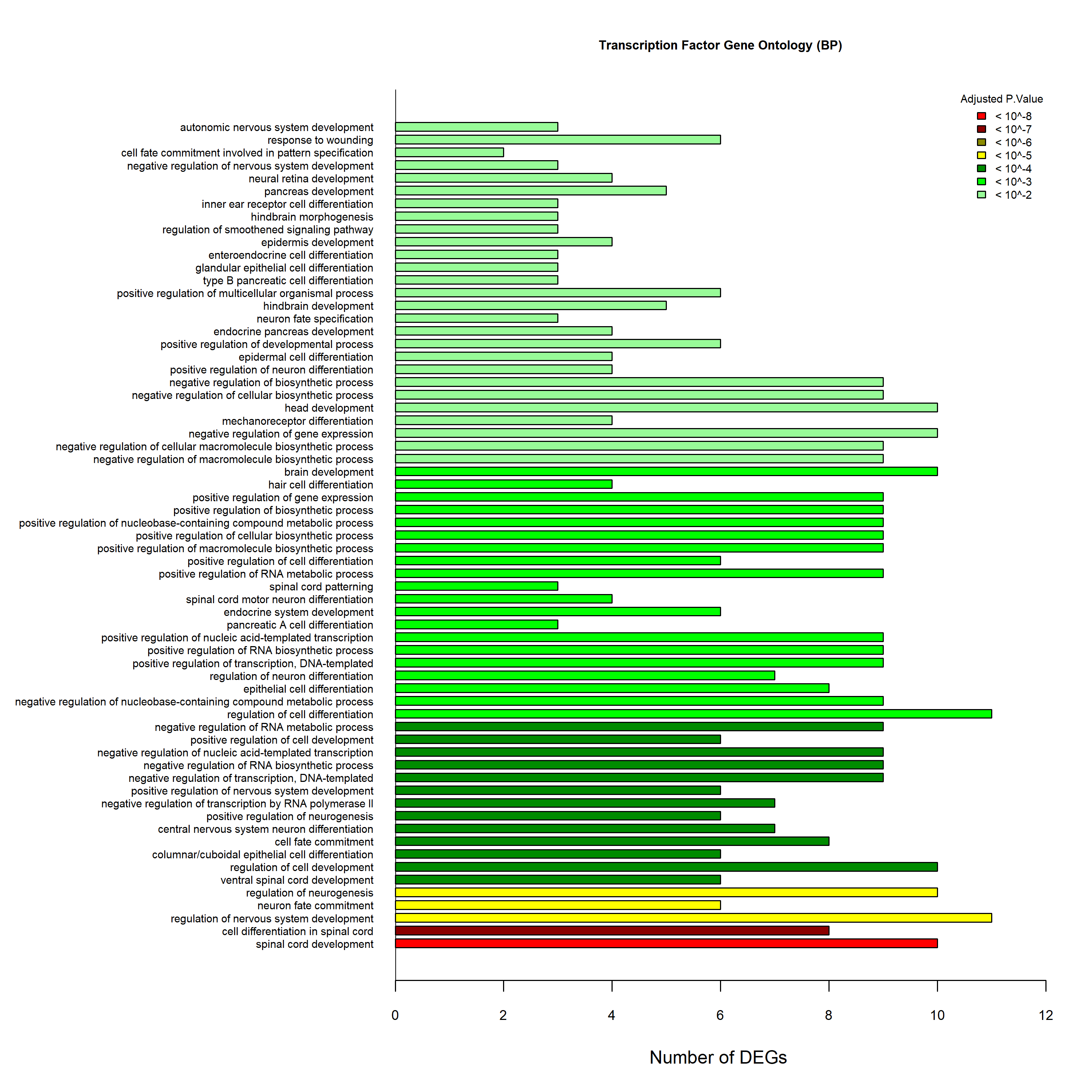

Supplement: Figure S3 — Barplot representing the number of differentially expressed transcription factors that map to each biological process gene ontology (P < 0.01). Transcription factors were derived from lepa knockdown compared to control. Color scheme reflects adjusted p.value for each ontology. [file peerj-07-6848-s003.png]

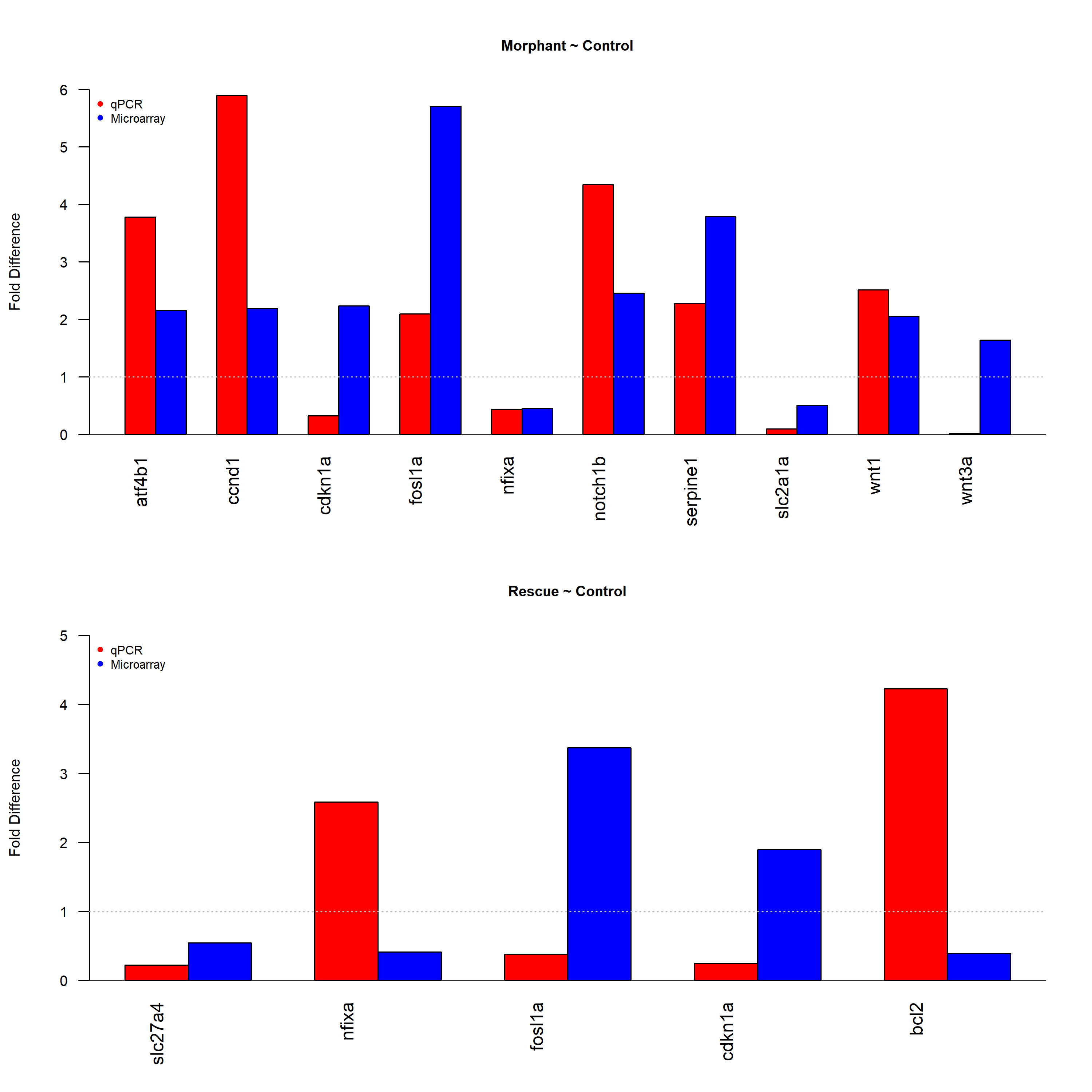

Supplement: Figure S4 — Barplot of qPCR data vs. microarray data for DEGs in the morphant vs. control comparison and the rescue vs. control comparison. A fold difference value of 1 indicates no change in expression; ¿1 indicates a positive change in expression (e.g. morphants express more than control), and ¡1 indicates a negative change (e.g. morphants express less than control). N=2 for qPCR data (treatment and control), n=4 for morphant and rescue microarray data, n=8 for control microarray data. [file peerj-07-6848-s004.png]
